# Computed Tomography Images under the Nomogram Mathematical Prediction Model in the Treatment of Cerebral Infarction Complicated with Nonvalvular Atrial Fibrillation and the Impacts of Virus Infection

**DOI:** 10.1155/2022/3950641

**Published:** 2022-03-27

**Authors:** Yi Zhu, Hai Cheng, Rui Min, Tong Wu

**Affiliations:** ^1^Department of Emergency, Geriatric Hospital of Nanjing Medical University, Nanjing 210024, China; ^2^Department of Cardiology, Suzhou Kowloon Hospital, Suzhou 215000, China

## Abstract

The aim of this work was to explore the effect of the nomogram mathematical model on the treatment of cerebral infarction complicated with nonvalvular atrial fibrillation (NVAF) and viral infection. The data were scanned by a circular trajectory fan beam isometric scanning mode system (scanning system), and the speckle noise of computed tomography (CT) images was smoothed by Lee filtering. 52 patients with postoperative recurrent viral infection (RVI group) and 248 patients without postoperative recurrent viral infection (NRVI group) were selected for retrospective analysis. The mathematical model curve was then analyzed through calibration plots and decision curves to predict the accuracy of the mathematical model. The results showed that the area under the receiver operating characteristic curve (AUC), sensitivity, specificity, and accuracy based on the training set were 0.7868, 0.7634, 0.6982, and 0.7146, respectively. The AUC, sensitivity, specificity, and accuracy based on the validation set were 0.7623, 0.7734, 0.6882, and 0.6948, respectively. There was no significant difference in the AUC between the two groups (*P* > 0.05), indicating that the nomogram mathematical prediction model had high repeatability. In conclusion, CT images based on the nomogram mathematical prediction model had good predictive ability in the treatment of cerebral infarction complicated with NVAF.

## 1. Introduction

Atrial fibrillation is a common persistent arrhythmia in clinical practice, and the stroke caused by it has the characteristics of high fatality rate and disability rate, especially in the elderly (10%) [1, 2]. Atrial fibrillation is not only an arrhythmia but also an important independent risk factor that causes cerebral infarction. The incidence and mortality of atrial fibrillation combined with cerebral infarction not only affect the survival of patients but also bring a heavy burden to the family [3, 4]. There are two types of atrial fibrillation, VAF and NVAF. NVAF is a common type of atrial fibrillation in clinical practice. It refers to atrial fibrillation without rheumatic heart disease, artificial valve replacement, or valve repair. Patients with NVAF have a higher risk of thromboembolism and are prone to merging cerebral infarction [5–7]. With the progress of pathophysiology research in the field of atrial fibrillation, various mechanisms such as ion channel mutation and myocardial fibrosis have been proved to be independent risk factors for atrial fibrillation. Viruses such as *Helicobacter pylori* (HP) and herpes simplex virus (HSV) can induce a large amount of release protein C from the body and trigger an inflammatory response, thereby increasing the risk of atrial fibrillation [8]. There are also some studies that only diagnose viral infection based on previous medical history, lacking the corresponding etiological evidence. Infections of viruses such as macrophage virus (MV), Coxsackie virus (CoxV), and parvovirus (PV) are all related to incidence of cardiovascular disease (CVD), but the relationship between the inflammatory responses caused by these viruses and the atrial fibrillation is still inconclusive [9]. In recent years, the prevention of stroke in patients with NVAF has received high attention, but the effect is not obvious. Because of the hidden nature of atrial fibrillation, real-time monitoring of patients has become a trend. With the development of big data technology, the collection of monitoring data of patients with atrial fibrillation can better help doctors summarize the characteristics of the disease, laying the foundation for the follow-up implementation of personalized treatment [10].

CT is one of the important bases for diagnosing cerebral infarction. Cerebral infarction can show abnormalities on CT images for 3–6 hours, mainly including increased aortic density [11, 12]. Although radiofrequency ablation and surgical management during the surgery have improved the survival rate after atrial fibrillation, the recurrence rate caused by viral infection still is not eliminated [13, 14]. Early recurrence prediction tools are still relatively lacking. A tool is needed for personalized assessment of recurrence caused by viral infection, and effective treatment measures can be taken by physicians to assess the probability of early postoperative recurrence [15, 16]. Mathematical models can make quantitative predictions based on the occurrence of diseases. For some specific objects, the mathematical models can be approximated according to the existence of some risk factors and expressed in mathematical structures, and then the statistical language and programming calculations can be adopted to make batch predictions. At this stage, there are mainstream methods of disease prediction, and these have become very popular methods for disease control and health policies. The American Heart Association (AHA) has used the establishment of CVD prediction models to propose prevention strategies, and many researchers have proposed prevention strategies for infectious disease prediction models. The application of mathematical prediction models is becoming more and more popular in medicine [17]. On the basis of multifactor analysis, a model that integrates multiple predictive indicators and predicts the probability of an outcome event individually and accurately has achieved lots of predictive effects in many diseases [18].

Therefore, in this experiment, the nomogram mathematical model was adopted to predict the probability of recurrence of atrial fibrillation after postoperative viral infection, so as to study the influencing factors of treatment of cerebral infarction combined with NVAF and postoperative viral infection. The individualized mathematical model could provide valuable information for clinical treatment. Statistical methods combined with computer software analysis finally got the experimental results. It was hoped that the application of the mathematical model in this study could provide an effective basis for evaluation of disease prevention, policy decision-making, and the effect of health intervention programs, so as to achieve the rational use of medical resources.

## 2. Methods

### 2.1. Research Objects

**Figure 1:**
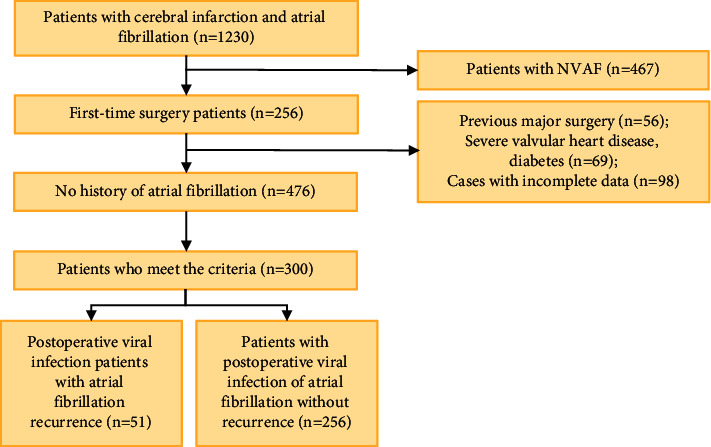
The screening process of research objects.

Patients with cerebral infarction combined with VAF who were admitted to hospital from June 2018 to June 2020 were selected as the research objects in this study. The family members of the patients all were given informed consent and signed the unified informed consent. This study had been approved by the Ethics Committee of the hospital. The screening process of research objects is presented in Figure 1.

*Inclusion criteria*. The diagnostic criteria of acute ischemic cerebral infarction were referred to the Chinese Acute Ischemic Stroke Diagnosis and Treatment Guidelines 2018: patients suffered from acute onset, neurological deficits, and ischemic lesions shown by imaging examination caused by nonvascular causes; patients with atrial fibrillation proved by the electrocardiogram (ECG) and no heart valvular disease proved by heart color Doppler ultrasound; patients whose onset was less than 72 hours; and patients who voluntarily participated in this research. According to the American College of Cardiology Atrial Fibrillation Management Guidelines 2014, the atrial fibrillation was defined as a supraventricular tachyarrhythmia characterized by the electrocardiogram, disappeared P wave, appeared irregular atrial fibrillation waves, and absolutely irregular R-R interval.

*Exclusion criteria*. Patients with severe organ dysfunction; patients with transient ischemic attack without infarction; patients with motor, sensory, language, or higher cerebral cortical dysfunction caused by other noncerebrovascular diseases; patients without vascular causes; patients who were critically ill and could not cooperate to improve relevant auxiliary examinations; and patients who had received other treatments within three months.

Fifty-two patients with postoperative recurrent viral infection (RVI group) were selected, including 30 males and 22 females, aged 32–63 years, with an average age of 52.01 ± 7.08 years. 248 patients without recurrent viral infection after surgery (NRVI group) were selected, including 130 males and 118 females, aged 31–63 years, with an average age of 52.01 ± 8.13 years. The research objects were retrospectively analyzed, and all patients underwent CT scan. The oral hematemesis and clinical baseline of all patients who met the inclusion criteria and did not meet the exclusion criteria should be collected within 24 hours of admission, and their diagnosis and treatment information during the hospitalization had to be completed within 24 hours of discharge or death. After screening, the clinical data of all eligible patients were recorded, including database number, admission time, gender, age, hospitalization number, and onset time. CT examination was performed for the heads of all patients.

According to the patient demographic data and entries on the CHA_2_DS_2_-VASC scale and HAS-BLED scale, the following factors of patients were observed. Congestive heart failure (CHF): the patient suffered from a clear history of CHF, was diagnosed as heart failure during the hospitalization, and was confirmed by the ECG to meet the Boston diagnostic criteria. Diabetes: the patients had a clear history of diabetes (treated by oral hypoglycemic drugs or insulin), previous bleeding or bleeding tendency, and hemorrhagic stroke.

The patients in the observation group and the normal control group were descriptively analyzed. The distribution of each related factor in the cohort was calculated and performed with the single-factor analysis to clarify the statistical difference of each related variable in the distribution. *T* variable was applied for the linear variables, and the chi-square test was applied for the nonlinear variables. The CHF, hypertension, age ≥75 years, diabetes, and stroke/transient ischemic attack (CHADS_2_) were scored from 5 indicators, as shown in Table 1.

### 2.2. Logistic Multivariate Regression Analysis

Logistic multivariate regression analysis was based on the premise of single-factor analysis to find relevant independent risk factors. The selection of independent variables of the logistic multivariate regression mathematical model was mainly based on Akaike information criterion (AIC) statistics.

The AIC was a trade-off between the complexity of the estimated model and the goodness of the model to fit the data, and it was a standard to measure the goodness of the statistical model. The calculation equation was AIC = 2k − 2ln(L), of which K represents the amount of model fitting parameters and *L* refers to the likelihood function.

Increasing the number of fitting parameters could effectively improve the goodness of model fitting, but it was prone to overfitting. The minimum AIC value was determined to select the fitting parameters and the corresponding model, which could effectively avoid overfitting when the model was excellent. AIC could find the model with the best interpretation of the data but with few free parameters.

### 2.3. CT Image Processing

**Figure 2:**
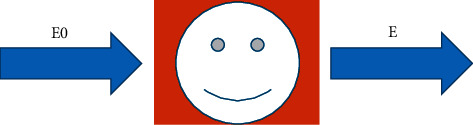
The schematic diagram of Beer's law.

Circular artifacts were more common in CT images, which were mainly due to the inconsistency of the detectors. There were many methods for image processing in the mathematical model, such as multifocus image fusion, Fourier transform, or high-pass filtering. The mathematical model of CT projection data was Radon transformation, which was actually some line integral values. The projection data were determined by the intensity of the object's ray, and the intensity was also related to the material composition of the object. If the initial intensity of the X-ray incident was E0, a binary function can be undertaken as the distribution of a certain fault of the detected object, and the emission intensity of the ray after passing through the object was E. The schematic diagram is shown in Figure 2.

According to Beer's law, the relationship between the initial intensity of the ray and the intensity of the ray was as follows:

(1) $$\begin{array}{c} E\left(L\right)=E_{0}\exp\left\{-\int_{L}\text{f}\left(x\right)\text{d}l\right\}. \end{array}$$

In equation (1), *L* represents the line where the ray lies, and d*l* was the integral element of the line. In equation (1), if r was expressed x, then the vector = (cos, sin), and the angle was the counterclockwise formed by the x1 axis and the normal of the straight line, r referred to the distance from the straight line to the origin of the coordinate. Then, the below equation could be obtained:

(2) $$\begin{array}{c} P\left(\delta ,\text{r}\right)=-\text{In}\left(\frac{E}{E_{0}}\right)=\int_{LX\psi =r}\text{f}\left(x\right)\text{d}l. \end{array}$$

The Radon change of the function *f(x)* was *P*(*δ*, r), which refers to the integral of *f(x)* along the straight line *r* = xΨ. When *δ* was fixed, *P*(*δ*, r) was a projection of *f(x)*.

**Figure 3:**
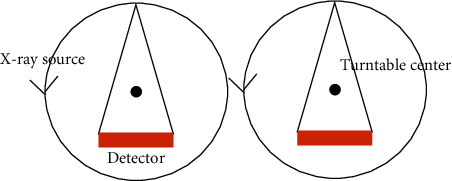
The schematic diagram of circular track fan beam isometric scanning mode.

In different scanning mathematical models, the projection data presented by the system were also different. In the current medical CT system, there was a spiral trajectory fan scan mode, and the original trajectory was equidistant. In this study, the circular track fan beam isometric scanning mode was used, and the schematic diagram of which is shown in Figure 3.

Random speckle noise was a limitation of ultrasound images, caused by the interference of uneven fine tissue scattering. Due to the presence of noise, the spatial resolution of the image was reduced, and the interpretability of the image was reduced. Speckle noise was not conducive to the recognition of the image by the naked eye, and it concealed the effective features extracted by the image, so it was particularly important to effectively denoise the image. A better image denoising model was adopted in this study, and speckle noise was the correlation between multiplicative noise and image signal:

(3) $$\begin{array}{c} f\left(a,b\right)=g\left(a,b\right)\delta m\left(a,b\right). \end{array}$$

In equation (3), *f(a, b)* represents the original image with noise, *g(a, b)* represents the uncertain noise-free image, and *δm(a, b)* refers to the multiplicative noise.

Image speckle denoising was also called multiplicative noise. Lee filtering was done to filter out image speckles based on the statistical characteristics of the image, select the length of the local area window on the speckle noise model, and obtain the prior mean and variance by calculating the local variance and mean.

(4) $$\begin{array}{c} \hat{\text{x}}=ax+by\\ \hat{\text{x}}=\bar{x}+b\left(y+\bar{x}\right)\\ a=1-\frac{\mathrm{var}\left(x\right)}{\mathrm{var}\left(y\right)}\\ \text{b}=\frac{\mathrm{var}\left(x\right)}{\mathrm{var}\left(y\right)}\\ var\left(\text{x}\right)=\frac{\mathrm{var}\left(y\right)-\beta_{y}^{2}y^{2}}{1+\beta_{y}^{2}}. \end{array}$$

**Figure 4:**
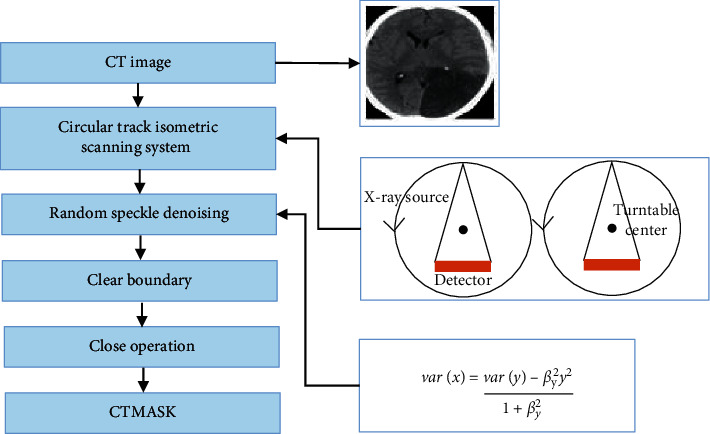
Preprocess flow of CT image.

Equation (3) assumed the form of a linear filter; $\hat{\text{x}}$ and $\bar{x}$ were the minimum mean square estimate and the average value of *x*, respectively; and var(y) referred to the variance. If var(x) approached 0, it meant that the local area was uniform, and $\bar{x}$ = $\hat{\text{x}}$ indicated the average pixel value in the window. If var(x) was relatively large, equal to *y*, it was the pixel value of the image itself, which meant that the noise in the marginal area could not be smoothed. The preprocess flow of the CT image is presented in Figure 4.

### 2.4. Model Evaluation

The AUC is defined as the area enclosed by the ROC curve and the coordinate axis, and the value of AUC ranges between 0.5 and 1. The closer the AUC is to 1.0, the higher the authenticity of the detection method. When it is equal to 0.5, the authenticity is the lowest. The recognition rate, sensitivity, and specificity were selected to evaluate the model construction, which were calculated using the below equations:

(5) $$\begin{array}{c} \text{Accuracy}=\frac{\left(TP+TN\right)}{\left(TP+TN+FP+FN\right)}\\ \text{Sensitivity}=\frac{TP}{\left(TP+TN\right)}\\ \text{Specificity}=\frac{TN}{\left(TN+FP\right)}. \end{array}$$

Here, TP is the true positive, FP is the false positive, TN is the true negative, and FN is the false negative.

### 2.5. Statistical Methods

SPSS 22.0 software was adopted to perform statistical processing of the experimental data. Quantitative data were expressed in the form of mean ± standard deviation ($\bar{x}\pm S\;D$), and the independent sample *t*-test was used for difference comparison. The binary variables were given in the form of percentage (%), and the difference was compared using the chi-square test. *P* < 0.05 indicates that the difference between groups was statistically significant. On the basis of Logistic regression, the incidence probability of postoperative atrial fibrillation was predicted by using a line map, and the prediction ability and discrimination performance of the line map were evaluated. A consistent C-index Hull calibration curve was constructed. The larger the index, the more accurate the predicted value. The net income was quantified under different threshold probabilities to determine the clinical utility of the nomogram and verify the radical internal verification.

## 3. Results

### 3.1. Basic Data of Research Objects

The patient was followed up by telephone for three months after the onset of the disease to understand their quality of life in time, which was assessed using the modified Rankin Scale (mRS). The age, gender, coagulation function, cerebral infarction area, history of alcohol abuse, and CHF of the patients were assessed after admission, as shown in Table 2.

### 3.2. Analysis of Clinical Characteristics of the Two Groups

While the independent variables of the logistic multivariate regression model were selected, the Pearson correlation test can also be performed on the corresponding independent variables. If the two independent variables were statistically significantly correlated, the two independent variables and their interaction variables were put into the model, and the independent variables in the mathematical model were finally selected by AIC. It was necessary to further evaluate the nonlinearity of linear variables such as age to determine whether it was more suitable for the group model. The percentile was used to classify linear variables into nonlinear variables, which was subjective, so that some important information was lost. Therefore, the use of restricted cubic splines had a very good performance for the fitting of the model. The results of logistic multivariate regression analysis on the clinical characteristics between patients in the RVI group and NRVI group after atrial fibrillation are provided in Table 3.

### 3.3. Example of Nomogram

**Figure 5:**
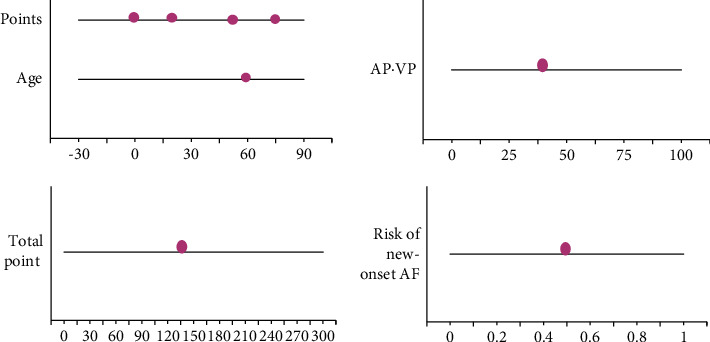
Nomogram figure of a patient with cerebral infarction combined with NVAF.

A 60-year-old patient was selected as an example. As illustrated in Figure 5, the corresponding score for the age variable was 50 and the score for peripheral arterial disease was 70. If the left atrium diameter increased by 43 mm when the patient was hospitalized, the corresponding score for the left atrium was 25. In the postoperative follow-up, if the AV-VP was less than 50%, the corresponding score of AP-VP was 0, and the sum of the above was 145. Analysis of the corresponding data on the total score axis and the new-onset AF axis showed that the risk of recurrence of atrial fibrillation was 50%.

### 3.4. Verification of Prediction Model

**Figure 6:**
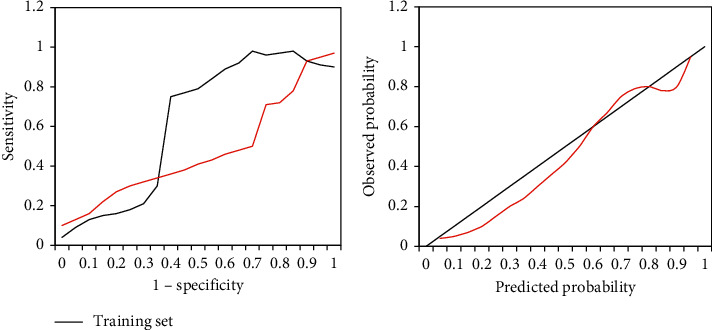
The ROC curve and model calibration curve of the prediction model on the training set and validation set.  ^*∗*^ indicates the difference was statistically obvious (*P* < 0.05).

The prediction model showed higher effects on the recurrence of atrial fibrillation caused by viral infection on both the training set and the validation set (as shown in Figure 6). The AUC, sensitivity, specificity, and accuracy on the training set were 0.7868, 0.7634, 0.6982, and 0.7146, respectively; and those on the validation set were 0.7623, 0.7734, 0.6882, and 0.6948, respectively. There was no visible difference in the AUC between the two groups (*P* > 0.05), indicating that the nomogram prediction model showed high repeatability. The model calibration training graph revealed that the graph was closer to the diagonal, indicating that the prediction was highly consistent with the observation result, so the model had a good predictive ability.

### 3.5. CT Images of Cerebral Infarction

**Figure 7:**
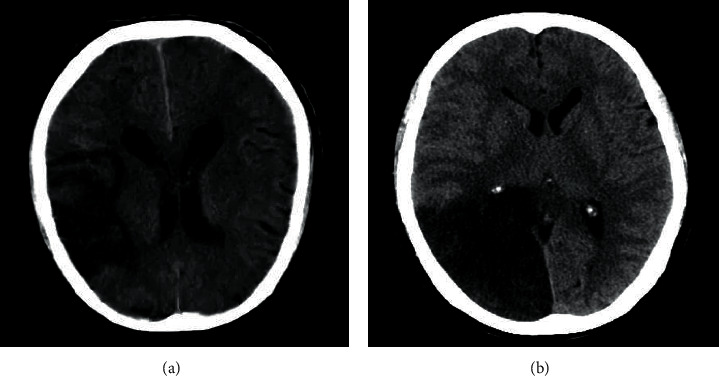
CT images of cerebral infarction.

Figure 7 shows the CT images of patients with cerebral infarction combined with NVAF after viral infection after surgery. The cerebral sulcus around the infarcted vessel was swollen, the lenticular nucleus was fuzzy, and the density of cerebrospinal fluid was greatly reduced.

### 3.6. The Clinical Value of Nomogram

**Figure 8:**
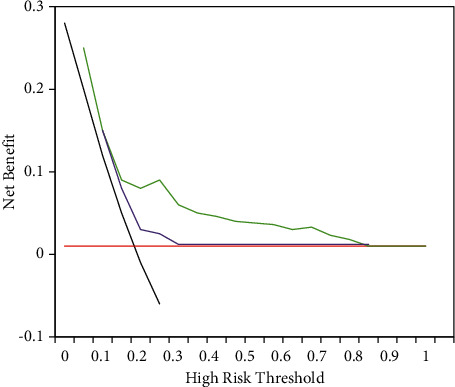
Clinical decision curve of the predictive model of atrial fibrillation after viral infection.

In clinical application, the clinical decision curve is a new method for evaluating the predictive models. The nomogram of atrial fibrillation caused by viral infection is shown in Figure 8. The purple solid line represents the nomogram model of new-onset atrial fibrillation after postoperative viral infection, the yellow dashed line represents the single-factor predictive model of AP-VP ≥ 50%, and the red solid line indicates that all patients had postoperative new-onset cerebral infarction combined with NVAF. The *X*-axis represents the valve probability, and the *Y*-axis represents the net benefit. The range of the probability of occurrence was within the range of 12%–40%. The clinical net benefit of the mathematical prediction model of the nomogram in the study was higher than that of the single-factor model of AP-VP ≥ 50% and left atrial diameter. Such results indicated that the mathematical prediction model of this study had clinical application value.

## 4. Discussion

By staining atrial tissue from patients with atrial fibrillation, the researchers found macrophages, high expression of tumor-transforming factors, and other inflammatory factors. Inflammatory factors interfere with cardiomyocytes and induce fibroblast activation. Serum levels in patients with atrial fibrillation are affected by cardiovascular disease. Viral infection leads to recurrence of atrial fibrillation, which is associated with local inflammation caused by viral invasion of myocardial tissue. Ikeda et al. (2019) [19] analyzed the dose of oral anticoagulant rivaroxaban in patients with NVAF, and different doses had different clinical treatments for patients. In patients with acute ischemic stroke and nonvalvular atrial fibrillation, pre-event DOAC treatment was associated with smaller infarct volume and a reduced risk of proximal large-artery occlusion compared with no anticoagulation. During the postoperative follow-up of the patients in this work, if the AV-VP was less than 50%, the corresponding AP-VP score was 0, and the sum of the above scores was 145. Analysis of the corresponding data on the total score subaxis and the new-onset AF axis showed a 50% risk of AF recurrence.

The nomogram is an analysis method that integrates multiple predictive indicators. After a multifactor regression analysis is performed, the same square meter is drawn with a reference line graph, which can predict the resolution of the indicators and clarify the relationship between the indicators. This method is quickly applied clinically and achieved ideal prediction results. Borumandnia et al. (2021) [20] applied the nomogram to the survival prediction evaluation of patients with rectal cancer, and the results showed that it can help patients with rectal cancer to solve clinical decision-making and prognosis prediction. Xia et al. (2017) [21] applied the nomogram mathematical prediction model to the prediction of cardiovascular disease in peritoneal dialysis patients, and the nomogram can accurately predict the cardiovascular death factors of patients. In this study, the existing medical records were reviewed and analyzed, the risk factors were explored, and a simple nomogram was established as a tool to predict the occurrence of atrial fibrillation in viral infections, whose effectiveness was verified accordingly. A mathematical model was constructed to analyze the characteristics of the CT images of patients. After sorting, the characteristics of the normalized image were extracted. The algorithm ignored the difference in image intensity and autonomously recognized the interpretability of ultrasound images for a more detailed analysis of the risk factors of atrial fibrillation in patients with viral infection. In this study, the prediction model showed higher effects on the recurrence of atrial fibrillation caused by viral infection on both the training set and validation set. The accuracy on the training set was 0.7146, and the accuracy on the validation set was 0.6948. There was no observable difference in the AUC between the two groups (*P* > 0.05), indicating that the nomogram prediction model had high repeatability. The model calibration training graph revealed that the graph was closer to the diagonal, indicating that it had better predictive resolution. The range of the probability of occurrence was within the range of 12%–40%. The clinical net benefit of the mathematical prediction model of the nomogram in the study was higher than that of the single-factor model of AP-VP ≥ 50% and left atrial diameter. Such results indicated that the mathematical prediction model of this study had clinical application value.

## 5. Conclusion

In this study, the existing medical records were reviewed and analyzed, the risk factors were explored, and a simple nomogram was established as a tool to predict the occurrence of atrial fibrillation in viral infections, whose effectiveness was verified accordingly. A mathematical model was constructed to analyze the characteristics of the CT images of patients, so as to analyze the risk factors of atrial fibrillation in patients with viral infection. The prediction model showed higher effects on the recurrence of atrial fibrillation caused by viral infection on both the training set and validation set. The new nomogram used in this study had a good predictive efficiency and could help physicians identify the risk of atrial fibrillation due to viral infection in the clinic. However, there were still missing values and erroneous data in the model data set in this work, and the imputation of the data in the later stage would affect the analysis results of the data. Further validation of the nomogram prediction model from different data sets is required in future research [2, 21].

## Figures and Tables

**Table 1:** Calculation methods of CHADS_2_ score and derivative scores.

CHADS_2_		CHA_2_DS_2_		R_2_CHADS_2_	
Risk factors	Score (s)	Risk factors	Score (s)	Risk factors	Score (s)
CHF	1	CHF	1	CHF	1
Diabetes	1	Diabetes	1	Diabetes	1
Age ≥75 years	1	Age ≥75 years	2	Age ≥75 years	1
Hypertension	1	Hypertension	1	Hypertension	1
Stroke/transient ischemic attack	2	Stroke/transient ischemic attack	2	Stroke/transient ischemic attack	2
		65 < age ≤ 75	1	Estimated glomerular filtration rate (GFR) ≤ 60	2
		Vascular disease	1		
		Female	1		
Total scores	6	Total scores	9	Total scores	8

Note: CHA_2_DS_2_ refers to CHF, hypertension, age ≥75 years [doubled], diabetes, stroke/transient ischemic attack. R_2_CHADS_2_ refers to renal dysfunction, CHF, hypertension, age ≥75 years, diabetes, stroke/transient ischemic attack.

**Table 2:** Basic data of research objects.

Factor	mRS ≤2	mRS >3	X^2^/*t*	*P*
Score of stroke at admission	9.00 (4.00, 11.00)	10.00 (7.00, 14.00)	−4.138	≤0.001
Age	68.72 ± 5.62	70.35 ± 5.81	−1.321	0.136
Coagulation function	1.07 ± 0.05	1.06 ± 0.03	−1.876	0.043
Gender	54	43	0.028	0.812
Cerebral infarction area	49	20	4.861	0.011
History of alcohol abuse	60	51	1.621	0.172
CHF	72	50	0.065	0.658

**Table 3:** Comparison of clinical characteristics between patients in the RVI group and NRVI group after atrial fibrillation.

Variable	Patients in the RVI group (*n* = 52)	Patients in the NRVI group (*n* = 248)	*P*
Diabetes (%)	11 (21.15)	48 (19.35)	0.287
Hyperlipidemia (%)	7 (13.46)	26 (10.48)	0.072
Diuretics (%)	6 (11.54)	21 (8.47)	0.154
Beta blockers (%)	7 (13.46)	27 (10.89)	0.132
Lipid-lowering drugs (%)	23 (44.23)	68 (27.42)	0.045
COPD (%)	2 (0.038)	13 (0.052)	0.218
Antiplatelet drugs (%)	19 (36.54)	72 (0.29)	0.467
Active atrial electrode (%)	28 (53.85)	145 (58.47)	0.523
Active ventricular electrode (%)	38 (73.08)	208 (83.87)	0.765
Cardiac ultrasound			
LVEF (%)	5 (9.62)	8 (3.23)	0.231
LAD (%)	21 (40.38)	61 (24.90)	0.056
Laboratory indicators			
BUN (mg/L)	5.26 (±1.86)	5.89 (±2.12)	0.248
C-reactive protein (mg/L)	1.5 (0.5–3.9)	1.5 (0.5–4.2)	0.543
Follow-up parameters			
AP-VP ≥ 50 (%)	11 (21.15)	19 (7.66)	0.012
AP ≥ 50 (%)	28 (53.84)	82 (33.06)	0.038
VP ≥ 50 (%)	29 (55.77)	89 (35.89)	0.069

Note: COPD refers to chronic obstructive pulmonary disease; LEVF refers to left ventricular ejection fraction; BUN represents blood urea nitrogen; VP refers to the score of sepsis-related organ failure assessment; and AP is the short form of adapted physical activity and cardiac coherence in hematologic patients (APACCHE).

## Data Availability

The data used to support the findings of this study are available from the corresponding author upon request.
